# Mueller Matrix Measurement of Electrospun Fiber Scaffolds for Tissue Engineering

**DOI:** 10.3390/polym11122062

**Published:** 2019-12-11

**Authors:** Dierk Fricke, Alexander Becker, Lennart Jütte, Michael Bode, Dominik de Cassan, Merve Wollweber, Birgit Glasmacher, Bernhard Roth

**Affiliations:** 1 Hannover Centre for Optical Technologies (HOT), Leibniz University Hannover, 30167 Hannover, Germany; lennart.juette@hot.uni-hannover.de (L.J.); m.wollweber@lzh.de (M.W.); bernhard.roth@hot.uni-hannover.de (B.R.); 2 Institute for Multiphase Processes (IMP), Leibniz University Hannover, 30167 Hannover, Germany; bode@imp.uni-hannover.de (M.B.); glasmacher@imp.uni-hannover.de (B.G.); 3 Institute for Technical Chemistry Technische Universität Braunschweig, 38106 Braunschweig, Germany; dominik@decassan.de; 4 Laser Zentrum Hannover e.V., 30419 Hannover, Germany; 5 Cluster of Excellence PhoenixD, Leibniz University Hannover, 30167 Hannover, Germany

**Keywords:** Mueller matrix, tissue engineering, electrospinning, fiber alignment, polycaprolactone

## Abstract

Electrospun fiber scaffolds are gaining in importance in the area of tissue engineering. They can be used, for example, to fabricate graded implants to mimic the tendon bone junction. For the grading of the tensile strength of the fiber scaffolds, the orientation of the fibers plays a major role. This is currently measured by hand in scanning electron microscope (SEM) images. In this work, a correlation between polarimetric information generated by measuring the Mueller matrix (MM) and the orientation of the fibers of electrospun fiber scaffolds is reported. For this, the MM of fiber scaffolds, which were manufactured with different production parameters, was measured and analyzed. These data were correlated with fiber orientation and mechanical properties, which were evaluated in an established manner. We found that by measurement of the MM the production parameters as well as the relative orientation of the fibers in space can be determined. Thus, the MM measurement is suitable as an alternative tool for non-contact, non-destructive determination of the production parameters and, thus, the degree of alignment of electrospun fiber scaffolds.

## 1. Introduction

**Figure 1:**
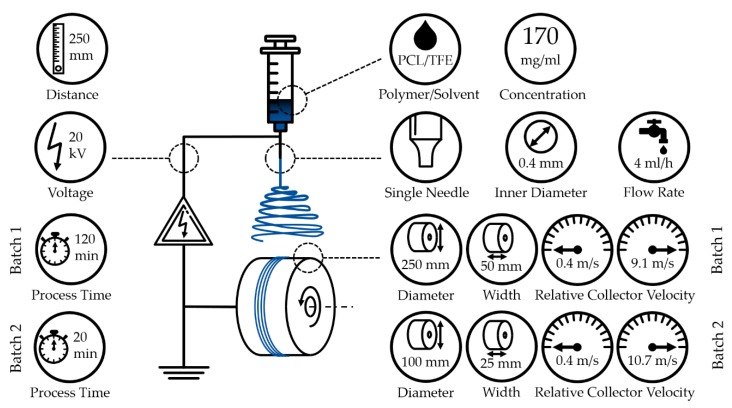
Schematic of the electrospinning device with manufacturing parameters. It consists of a syringe, a high voltage supply and a grounded collector. In addition, essential components/parameters (distinguished for both batches) are displayed: polymer, solvent, concentration, needle diameter, flow rate, collector to needle distance, used voltage, process time, dimensions of the collector and relative collector velocity.

The extracellular matrix (ECM) of biological tissues consists of different micro- and macroscopic fiber structures, depending on the tissue function. To recreate these tissues artificially, tissue engineering (TE) relies heavily on polymers as scaffold material to mimic the ECM [1,2,3,4,5,6,7,8,9,10,11]. The processing of polymeric solutions into fiber scaffolds by electrospinning has been carried out successfully. The manufactured fibers exhibit diameters between several hundred nanometers and a few micrometers. The basic electrospinning setup consists of an emitter, a high voltage supply and a grounded collector (see Figure 1). An initial droplet of the polymeric solution is vertically emitted into the electric field area generated by the high voltage supply. As a result of the forces induced by this field and gravity, the formation of the so-called Taylor cone is induced. Once the applied forces overcome the surface tension of the fluid, a fiber jet is emitted from the Taylor cone. While the fiber jet is constantly accelerated towards the grounded collector, the solvent starts to evaporate. At a certain point, bending instabilities occur, leading to a whipping movement of the fiber jet. As a result of the constant movement and solvent evaporation, the diameter is constantly decreasing upon deposition on the collector [2,12,13,14,15,16]. Different parameters like the humidity, temperature, partial pressure of the solvent, concentration of the polymeric solution, viscosity, and conductivity, as well as the surface tension of the solution, the emitter setup, the inner diameter of the emitter, the needle-to-collector distance, the flow rate, the geometry and relative collector velocity of the collector, and the applied electrical field and its distribution, along with the process duration, have a large influence on the electrospinning process [2,4,5,6,7,8,9,12,13,14,15,16,17,18,19,20,21,22,23]. The recreation of complex tissue structures like tendon–bone junctions call for graded implants with differently oriented fibers [1,10,24,25]. The fiber orientation within the manufactured scaffold is mostly influenced by the relative velocity of the collector: the higher the relative collector velocity, the higher the degree of fiber alignment [10,17,22,26]. A linear relation between relative collector velocity and degree of fiber alignment was hypothesized, as well as a linear relation between relative collector velocity and mechanical properties. It is also assumed that the mechanical properties for the different relative collector velocities show the same trend as the degree of fiber alignment.

The morphological characterization of the fiber scaffolds, usually relying on image analysis, is a potentially destructive, time consuming, expensive and inflexible process due to the nano- and micrometer-sized fiber structures. To determine the spatial orientation of electrospun fiber scaffolds, different methods and devices can be used, e.g., Raman spectroscopy [27], attenuated total reflection Fourier transform infrared (FTIR-ATR) spectroscopy [28] or scanning electron microscopy (SEM) [28,29,30,31,32,33,34]. However, commercially available Raman, FTIR or SEM devices are very expensive and relatively slow. Additionally, with these systems, only small increments of the fiber scaffolds can be measured, and representative analysis of the whole product is therefore very difficult and time consuming. Furthermore, the energy input occurring during the measurements, e.g., ionizing radiation, can cause an irreversible change of the polymer samples which would make them unsuitable for the initial purpose [3].

Similar to the energy input by ionizing radiation, sputter coating, a crucial step for SEM imaging, causes irreversible changes to the samples. During this process, a thin layer of metal is deposited on the fiber scaffold in order to enhance electrical conductivity. The actual morphological characterization is based on the evaluation of gray-scale images obtained with either one of the aforementioned technologies and conducted with an image analyzing software, e.g., ImageJ (manually), DiameterJ (automated) or AxioVision^®^ (manually) [26,28,29,30,31,32,33,34]. Ultimately, a method for the non-destructive, cost-efficient, reproducible and not locally limited morphological characterization is needed.

In this paper, we present a new method for measuring the orientation and manufacturing parameters of a fiber scaffold in non-contact and non-destructive mode. The used fiber scaffolds consist of polycaprolactone (PCL) fibers. This is realized by measurement of the Mueller matrix (MM) [35]. The latter is generally employed to describe the polarization changing properties of an object. Originally introduced in 1956 [36], the technology continues to attract interest in various research fields, especially in the field of tissue polarimetry, to investigate the orientation of structures like collagen [37,38,39]. In this context, it can also be applied to study human skin for different inflammatory skin diseases in dermatoscopy [40,41,42].

MM analysis of electrospun fiber scaffolds was previously shown by Wang et al. [43]. A microscopic setup was used [44,45] to gather information about the morphology of single fibers, e.g., the detection of embedded micropores or microspheres. Measurement times of 90 s in transmission mode and by using a single optical wavelength were reported. Here, we demonstrate large-area (several cm^2^) images of electrospun fiber scaffolds and determine the total and relative orientation of the fibers within about 15 s with the potential to measure even faster in an improved version. The setup presented can measure in transmission or reflection mode and with three different optical wavelengths, thus allowing for more flexible measurement applications in future.

Thus, with MM, the properties of fiber scaffolds can be determined quickly and non-destructively with previously impossible accuracy. Parameter of interest where the degree and direction of the alignment of the fiber scaffolds. To present and evaluate the MM measurement method, five scaffolds were produced and characterized using conventional characterization methods such as image analysis. Subsequently, the samples were also examined with MM and the results were discussed and evaluated. In a first step, the electrospun fiber scaffolds were analyzed with respect to fiber diameters and fiber alignment. The results shown in Section 3.1 are used to determine the influence of the relative velocity of the collector on these properties and at the same time investigate the possible impact on the mechanical properties and MM measurements. In Section 3.2, the correlation between the relative velocity and the mechanical properties of fiber scaffolds is discussed, as the latter are of particular interest for tissue engineering [11]. Finally, the fiber scaffolds were characterized by MM measurement and the results compared to the previous findings, as shown in Section 3.3.

## 2. Materials and Methods

### 2.1. Electrospun Fiber Scaffolds

#### 2.1.1. Processing System

The electrospinning device used in this work consists of a syringe, a syringe pump, polyethylene tubing, a blunt cannula, a high voltage supply, an electric motor and a drum collector, see Figure 1 and Table 1.

The syringe was mounted onto the syringe pump joint to the cannula via tubing. In addition, the cannula was electrically connected to the high voltage supply with the collector being the ground. By using a drill chuck, the collector was mounted onto the electric motor, thereby implementing the drum collector rotation. The circumferential surface of the collector was arranged perpendicularly in a vertical setup under the cannula.

#### 2.1.2. Parameter Settings and Experimental Procedure

The fabrication of the fiber scaffolds was conducted with a polymeric solution of polycaprolactone in 2,2,2-trifluoroethanol (see Table 1) with a concentration of 17% (*w*/*v*). Due to the different requirements of the analytical methods used, two different batches of fiber scaffolds were manufactured. If not stated otherwise, the fiber scaffolds were produced using a single-needle setup with an inner diameter of 0.8 mm, a needle-to-collector distance of 250 mm, a flow rate set to 4 mL/h and high voltage supply set to 20 kV while the current is 0 A. For damage-free removal of the fiber scaffolds, both collectors were covered with aluminum foil. The environmental parameters humidity and temperature were monitored. The first batch, consisting of 3 fiber scaffolds per relative collector velocity, was produced using a drum collector with a diameter of 250 mm and a width of 50 mm. Relative collector velocities of 0.4, 1,2, 2.0, 2.8, 3.6, 4.4, 5.2, 6.0, 6.7, 7.5, 8.3 and 9.1 m/s and a process duration of 120 min were set (see Figure 1 and Table 2). These parameters were chosen with regard to a certain fiber scaffold thickness which was necessary for the mechanical test samples. To achieve comparable results for both batches, the theoretical ratio of polymeric solution to drum collector surface (mL/mm^2^) was determined, using the values for batch 1 as the reference. Batch 2, on the other hand, with *n* = 5 per relative collector velocity, was manufactured by deploying a drum collector with a diameter of 100 mm and a width of 25 mm, relative collector velocities of 0,4, 1,2, 2.0, 2.8, 3.6, 4.4, 5.2, 6.0, 6.7, 7.5, 8.3. 9.1, 9.9 and 10.7 m/s and a process duration of 20 min (see Figure 1 and Table 2).

#### 2.1.3. Measurement of Fiber Alignment

**Figure 2:**
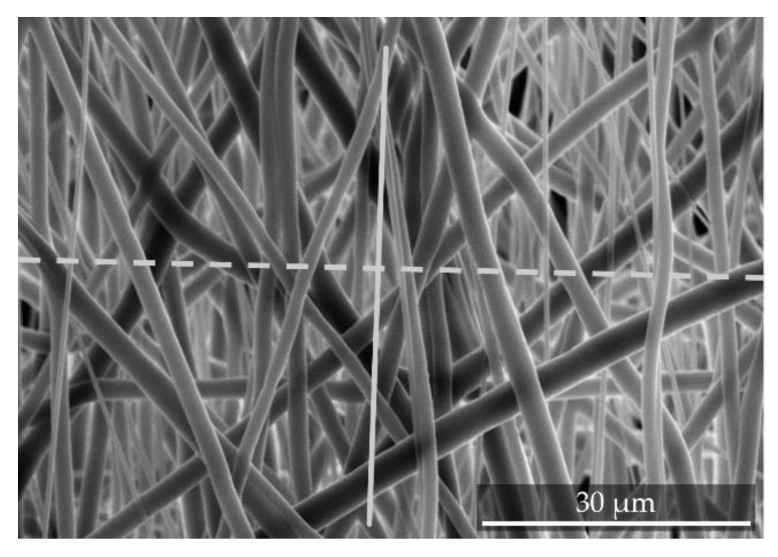
Exemplary SEM images of electrospun fiber scaffolds. For creating a reference for the analysis of fiber alignment, the (**solid**) line represents the moving direction of the collector. The second (**dashed**) line is perpendicular to the first line. To determine the orientation of each fiber, the angle between each fiber crossing the dashed line and this line is measured. The data shown is exemplary to further illustrate the method used.

To determine fiber alignment, SEM (S-3400N, Hitachi High-Tech Analytical Science Ltd., Tubney Woods, Abington, UK) images of 3 × 15 s sputter coated (SC7620, Quorum Technologies Ltd., Laughton, East Sussex, UK) samples were taken. Five images for each of the 12/14 (batch 1/batch 2) samples manufactured at different relative collector velocities were recorded. Subsequently, the images were analyzed by using the image analysis software (AxioVision^®^, Carl Zeiss AG, Jena, Germany). To assess the fiber alignment, each fiber scaffold sample was, prior to the imaging, folded in the direction of the relative collector velocity. These kinks were visible on the SEM images and a line was placed over them. A perpendicularly arranged second line was included in the images as well. The angle between every crossing fiber and this line was then analyzed (see Figure 2).

#### 2.1.4. Mechanical Testing

**Figure 3:**
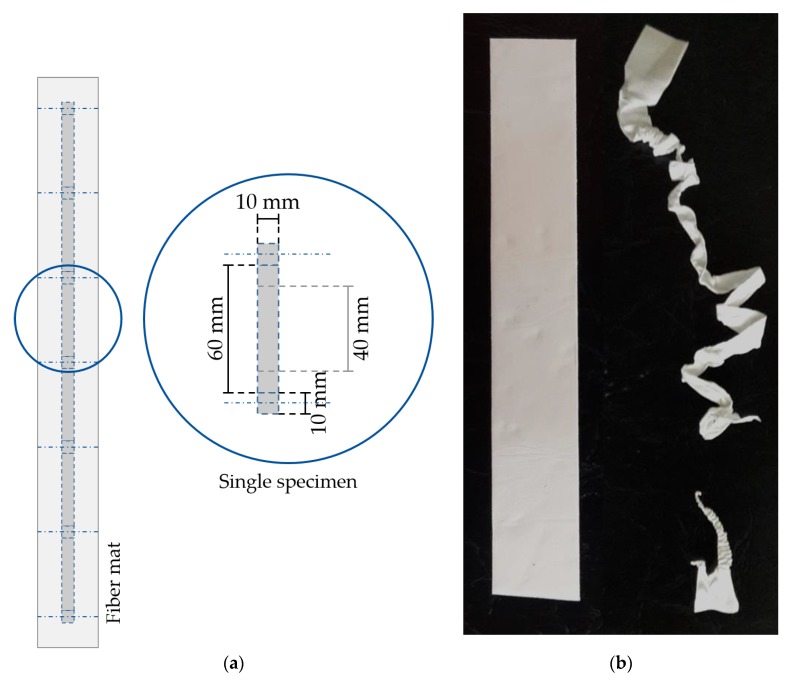
Procedure for the specimen preparation for tensile testing. Up to six samples were prepared from the middle of an electrospun fiber scaffold (**a**). Each sample exhibited a length of 60 to 70 mm, a width of 10 mm, and a gauge length of 40 mm. The specimen were mounted onto the testing machine and stretched until failure (**b**).

The reaction of the surrounding tissue to the scaffold depends on the scaffold properties, e.g., the mechanical properties [11,25,46]. Therefore, in order to determine the force at break and elongation at break, uniaxial tensile testing was carried out by employing a universal testing machine (5655, Instron, Norwood, MA, USA). Due to the necessary properties for tensile testing, e.g., stability during handling and mounting onto the machine, only samples from batch 1 were used. Three samples were prepared from the middle of each fiber scaffold for all 12 relative collector velocities. Each tensile testing sample displayed a width of 10 mm, a length of 60 to 70 mm and a gauge length of 40 mm (see Figure 3a). The specimens were mounted onto the testing machine using pneumatic grips. Each sample was tested until failure (see Figure 3b), with a crosshead speed of 40 mm/min and a 500 N load cell. These tests were conducted under room temperature. An alignment of the macroscopic fiber structure is assumed, as a response to external mechanical loads within scaffolds with low degree of fiber orientation.

### 2.2. Mueller Matrix Measurement System

The material properties of a sample can be deduced from its polarizing properties which is known in the literature for over 200 years [47]. Polarimetric measurement is used, for example, the determination of photoelasticity to investigate stress distribution in transparent bodies. The Mueller matrix (MM) provides access to information on how the sample interacts with light of different polarizations. The method of MM measurement is therefore used in the following to deduce the material properties of the fiber scaffolds.

#### 2.2.1. The Mueller Matrix

The MM formalism is connected to the Stokes formalism which describes the polarization state of light but not its phase information [48]. If the MM ($M_{m}$) of a sample is known, it is possible to calculate the Stokes vector of the outgoing light (${\vec{S}}_{o}$), after interaction with the sample, for every incoming light state (${\vec{S}}_{i}$) by:

(1) $$\left(\begin{array}{c} {\vec{S}}_{o1}\\ {\vec{S}}_{o2}\\ {\vec{S}}_{o3}\\ {\vec{S}}_{o4} \end{array}\right)\;=\;\left(\begin{array}{cccc} M_{11} & M_{21} & M_{31} & M_{41}\\ M_{12} & M_{22} & M_{32} & M_{42}\\ M_{13} & M_{23} & M_{33} & M_{43}\\ M_{14} & M_{24} & M_{34} & M_{44} \end{array}\right)\cdot \left(\begin{array}{c} {\vec{S}}_{i1}\\ {\vec{S}}_{i2}\\ {\vec{S}}_{i3}\\ {\vec{S}}_{i4} \end{array}\right).$$

The 4 × 4 MM therefore contains the information about the polarization changing properties of the sample. These properties are linked to its physical conditions.

The Stokes vector consists of the intensity values of different polarization states:

(2) $${\vec{S}}_{Stokes}\;=\;\left[\begin{array}{c} I_{H}+I_{V}\\ I_{H}-I_{V}\\ I_{P}-I_{M}\\ I_{R}-I_{L} \end{array}\right]\;=\;\left[\begin{array}{c} I_{H}+I_{V}\\ I_{H}-I_{V}\\ 2I_{P}-(I_{H}+I_{V})\\ 2I_{R}-(I_{H}+I_{V}) \end{array}\right],$$

where the different indices for the intensity *I* represent the values for the direction of the respective polarization states of the light (H stands for Horizontal polarization, *V* for Vertical, P for light that is polarized at an angle of 45°, M for light polarized at −45°, *R* for right and *L* for left circular polarization). The directions of polarization are defined in the coordinate system of the measurement setup and they are parallel to the sample plane. As seen in Equation (2), a Stokes vector can be determined by measuring the intensity of four different polarization states. If the MM is to be determined, it is necessary to measure the transformation of the incoming light state after interaction with the sample. Therefore, for every incoming intensity state, the outgoing intensity needs to be measured. This leads to at least 16 measurements to determine the MM. If six intensities for every Stokes vector are measured, the system is over determined and 36 different intensities need to be determined for the MM. In practice, this over determination leads to a compensation of calibration and measurement errors which increases the accuracy of the measurement.

The experimentally measured MM is not directly connected to a physical property of a measured sample. To interpret the MM, a decomposing is usually performed. Lu and Chipman proposed a polar decomposition where the MM is decomposed as follows [49]:

(3) $$M_{exp}=\;M_{\Delta }\cdot M_{R}\cdot M_{D}.$$

Here, $M_{exp}$ stands for the experimentally measured MM, $M_{D}$ for a diattenuator, $M_{R}$ for a retarder and $M_{\Delta }$ for a depolarizer. The form of these matrixes is known. For example, the depolarization matrix can be written as

(4) $$M_{\Delta }=\left[\begin{array}{cccc} 1 & 0 & 0 & 0\\ 0 & a & 0 & 0\\ 0 & 0 & b & 0\\ 0 & 0 & 0 & c \end{array}\right],$$

with condition |a|,|b|,|c|≤1 Also, some key figures can be calculated [49] to access specific properties of the sample such as the parameter Δ, which describes the depolarization power,

(5) $$\Delta \;=\;1-\frac{\left|a\right|+\left|b\right|+\left|c\right|}{3},0\leq \Delta \leq 1,$$

the parameter R, which describes the total retardance (the combination of the effect of linear and circular birefringence)

(6) $$R\;={\;\cos}^{-1}\left[\frac{tr\left(M_{R}\right)}{2}-1\right],$$

and the total polarizance *P* of an MM

(7) $$P\;=\;\frac{1}{M_{11}}\sqrt{M_{21}^{2}+M_{31}^{2}+\;M_{41}^{2}}.$$

While R ranges from 0 to π, P and Δ have values in the range from 0 to 1. It is also possible to calculate the normalized Stokes vector for the fast axis of the retardance *R*, from $M_{R}$ [49]

(8) $${\left(1,a_{1},a_{2},a_{3}\right)}^{T}={\left(1,{\hat{R}}^{T}\right)}^{T},$$

with

(9) $$a_{i}=\frac{1}{2\sin R}\sum_{j,k=1}^{3}\varepsilon_{ijk}{\left(m_{R}\right)}_{jk}$$

where $\varepsilon_{ijk}$ is the Levi-Civita symbol and $m_{R}$ is a submatrix of *M* without the first column and row.

The polar decomposition is not symmetric and, therefore, even the order of the elements $M_{D},\;M_{R}$ and $M_{\Delta }$ influences the result [50,51]. That is why other approaches rely on symmetric or sum decomposition, for example, which are independent of the order of the elements [48,52,53,54,55,56]. However, the polar decomposition is widely used in the literature [54,57,58,59,60], and also in this work.

#### 2.2.2. Mueller Matrix Imaging

**Figure 4:**
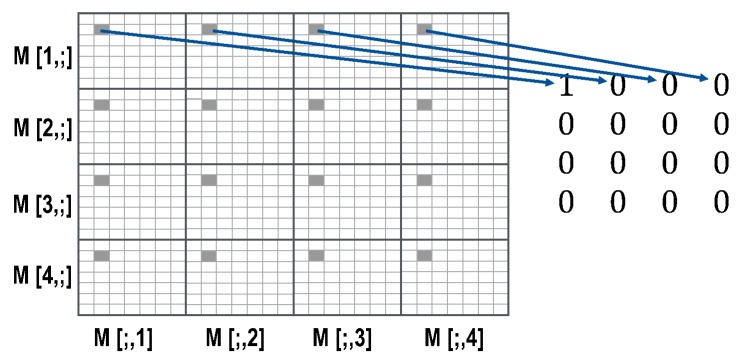
Visualization of the concept of spatially resolved MM. The result of the MM imaging is a 4 × 4 image matrix. The MM is determined for every pixel of the image. Therefore, spatially resolved MM information can be displayed, see an example representation for a single camera pixel on the right. The example MM on the right side shows the MM of an ideal diffusor for the specific pixel imaging a point of the sample. One sample image (e.g., *M*[2;2]) displays in every pixel the result for the assigned MM entry (e.g., *M*22). This gives, for example, information about the homogeneity of the structure of a given sample.

A black and white image can be seen as 2D intensity information. A camera measures the intensity for different areas of an object, and then gives quantified information depending on pixel size and bit depth. The outcome is an intensity matrix with size depending on number of pixels of the sensor. With MM imaging, we obtain a 4 × 4 matrix of this image-sized matrix of a sample, as shown in Figure 4. The pixel information for each of the submatrices of the 4 × 4 matrix represents the value of the specific MM element in the area imaged by the particular pixel.

To calculate the MM from the intensity information, at least 16 different measurements are required. In the case that calibration errors of the system need to be compensated, 36 different measurements are recommended. For MM imaging, this means that 36 images must be taken, with different polarization states of illumination and detection. After the images are taken, the different MM matrix entries are calculated for each pixel, see Table 3:

To display the image data, all MM images are normalized by the first image matrix element *M*[1,1]. Because of that, every MM value is ranged between −1 and 1.

#### 2.2.3. The Mueller Matrix Measurement System

**Figure 5:**
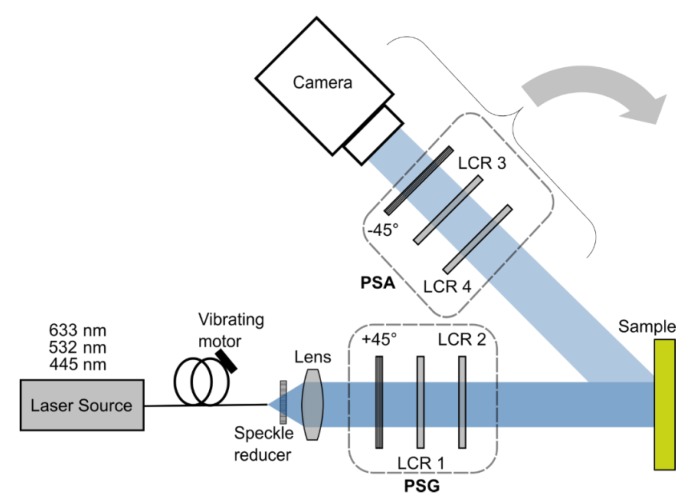
Experimental MM measurement system. It can measure the location-resolved MM of a sample for three different wavelengths. The laser sources can be switched by connecting different fibers. Because the LCRs require monochromatic light, speckles usually occur. To reduce their influence, the fibers are modulated by a vibrating motor connected to them. The light that exits the fiber is then collimated by a lens and passes the polarization state generator (PSG) consisting of a linear polarizer and two LCRs. By switching the voltage of the LRCs, different polarization states can be generated. After the light interacted with the sample, it passes the polarization state analyzer (PSA), which is essentially a PSG but with elements in the opposite order, to detect a specific polarization state. The light is then measured by a camera which generates a 2D intensity image. The analyzer arm can also be rotated to realize another scattering angle or to measure in transmission mode.

As explained above, to measure the MM, different polarization states must be generated and measured. With the system presented here, it is possible to measure the intensities required to calculate the MM out of 16 or 36 different measurements. For this, all polarization states given in Equation (2) and Table 1 must be generated [42]. As the system was developed for in vivo MM measurement, it acquires the images comparably fast within 20 s (depending on the intensity of the light source). In order to achieve this, the system does not contain moving parts and can generate all states electronically using liquid crystal retarders (LCR). The measurement setup is shown in Figure 5.

To display the results, the average values were calculated over image sections in which the sample is homogeneous. For samples which are produced under the same conditions, a measured value is determined for which a standard deviation is calculated according to:

(10) $$\sigma \;\sqrt{\frac{\sum^{\;}{\left(x-\bar{x}\right)}^{2}}{\left(n-1\right)}}\;,$$

where $\bar{x}$ is the mean sample value and *n* is the sample size.

Three different laser sources are used: A 633 nm source (HeNe, 25-LHR-991-230, CVI Melles Griot GmbH, Bensheim, Germany), a 532 nm source (CW532-04-1, ROITHNER LASERTECHNIK GmbH, Vienna, Austria) and a 445 nm source (LDMF series VLD-XT 445100, LASOS Lasertechnik GmbH, Jena, Germany). These are coupled into a multimode fiber (FT030, Thorlabs, Newton, NJ, USA) to which a vibrating motor (VM-0610A3.0, EKULIT Elektrotechnik Karl-Heinz Mauz GmbH, Ostfeldern, Germany) is attached, which is used to reduce the speckles by modulating in time. At the end of the fibers, an additional speckle reducer is attached (LSR-3010 Series, Optotune, Diekiton, Switzerland), which also modulates the speckles in time with a frequency of about 100 Hz. The beam is collimated and enters the polarization state generator (PSG), which consists of a linear polarizer (LPVISE100-A2", Thorlabs, Newton, NJ, USA) and two LCRs (LCC1221-A, Thorlabs, Newton, NJ, USA). The fast axis of the LCR 1 is oriented at 0° and the fast axis of the LCR 2 at 122.5°. With this constellation the PSG can generate all polarization states needed to measure the 36-image MM. After interaction with the sample, the light passes the polarization state analyzer (PSA) which consists of the same components as the PSG but in the opposite order. The location-dependent intensity measurement is then performed by a monochrome camera (BFS-U3-32S4M-C, FLIR Integrated Imaging Solutions Inc., Richmond, British Columbia, Canada) with a resolution of 2048 × 1536 pixel at a pixel size of 3.45 µm and a frame rate of 118 fps.

Depending on the light power used, the system can take images for the full 36-image MM in about 15 to 20 s. The delay between measurements is most likely due to the communication protocol of the LCR drivers. The data is displayed as a matrix of images, see Figure 4 and Figure 13 and discussion.

## 3. Results

### 3.1. Fiber Orientation

**Figure 6:**
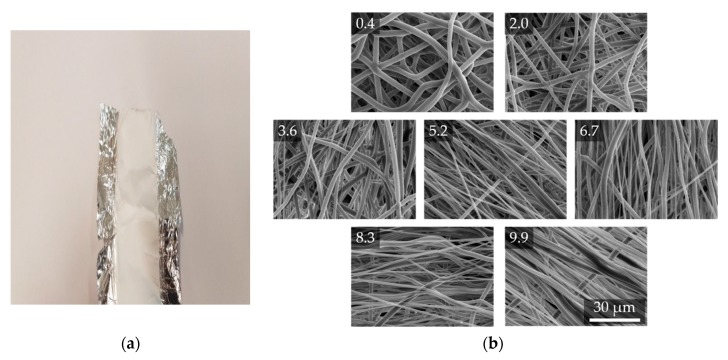
Exemplary image of a fiber scaffold on aluminum substrate lying on a white paper background (**a**). SEM images of fiber scaffolds for seven different relative collector velocities from batch 2; employed velocities from top left to bottom right: 0.4, 2.0, 3.6, 5.2, 6.7, 8.3 and 9.9 m/s, (**b**).

The manufactured fiber scaffolds for the different relative collector velocities applied are not distinguishable by the eye. They appear homogeneous and smooth (see Figure 6a). To analyze fiber diameters (see Supplementary Materials) and orientation, SEM images were used to determine the microscopic morphology (see Figure 6).

The morphological investigation, for batch 1 and batch 2, showed no linear relation between relative collector velocity and fiber diameter. Furthermore, a similar trend was observed for batch 1 and batch 2.

**Figure 7:**
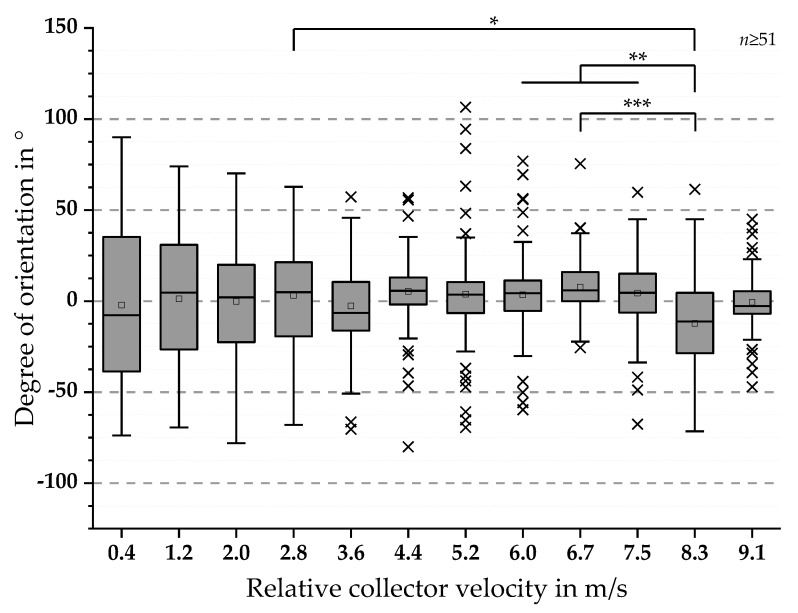
Boxplots of the fiber orientation in ° with respect to the collector’s moving direction for batch 1. The results show boxplots with decreased dispersion of the values with increasing relative collector velocity. Normal distribution for all data sets were found via Shapiro-Wilk and Kolmogorov-Smirnov-tests. Statistical significances were analyzed for all groups via one-way ANOVA test. The differences between the groups are displayed and labeled as follows: *** *p*-value < 0.001, ** *p*-value < 0.01 and * *p*-value < 0.05.

The SEM-based analysis for batch 1 resulted in degrees of orientation between −80° and 106.5° with calculated mean values alternating around 0°. The results exhibit decreased dispersion of the angular orientation values with increasing relative collector velocity (see Figure 7).

Furthermore, the results for the analysis of the fiber orientation for batch 1 are shown in Figure 7. Degrees of orientation between −80° and 106.5° were detected with calculated mean values alternating around 0°. Despite this observation, a distinct decrease in the dispersion of the values is observable for increasing relative collector velocity. For statistical proof of this observation, again, Shapiro-Wilk and Kolmogorov-Smirnov tests were conducted, indicating normally distributed data sets. Based on these results, a one-way ANOVA test was conducted, showing significant differences in degree of fiber orientation for 2.8 m/s and 8.3 m/s (* *p*-value < 0.05), 6.7 m/s and 8.3 m/s (*** *p*-value < 0.001) as well as 6.0 m/s to 7.5 m/s and 8.3 m/s (** *p*-value < 0.01).

**Figure 8:**
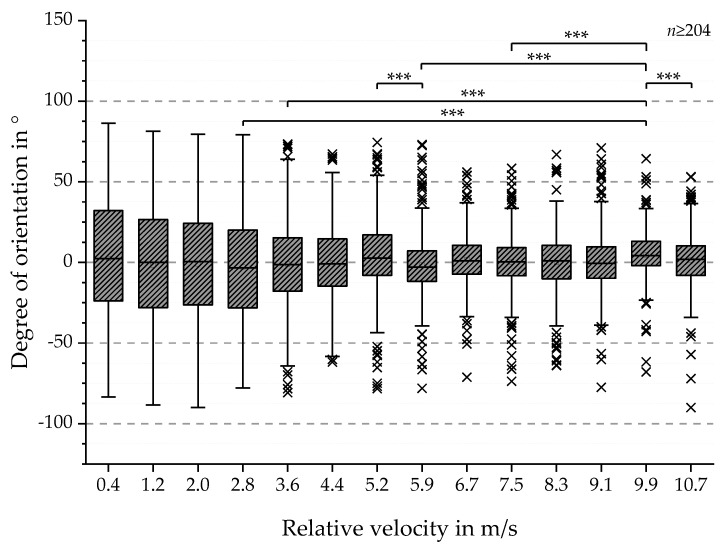
Boxplots of the degree of orientation in ° with respect to the relative collector velocity for batch 2. The results show boxplots with decreased dispersion of the angular orientation values with increasing relative collector velocity. Normal distribution for all data sets were rejected via Shapiro-Wilk and Kolmogorov-Smirnov tests. Statistical significances were analyzed for all groups via a Kruskal-Wallis ANOVA test followed by a Mann-Whitney test. The results with the highest *p*-value (*** <0.001) are displayed.

In addition, the degree of orientation for batch 2 was also investigated based on SEM images. The results are shown in Figure 8 and exhibit a range from −90° to 86.3° and calculated mean values around 0°. Similar to the observations for batch 1 (see Figure 7), the dispersion of the values decreases with increasing relative collector velocity (see Figure 8). For the purpose of statistical proof of this observation, Shapiro-Wilk and Kolmogorov-Smirnov tests were conducted, resulting in not normally distributed data sets. Based on these results, a Kruskal-Wallis one-way analysis of variance (ANOVA) test was conducted, indicating significant differences in the degree of fiber orientation. Therefore Mann-Whitney tests were executed, resulting in a vast amount of significantly different groups. The groups with the highest differences in degree of orientation (*p*-value 0.001 (***)) are 2.8 and 9.9 m/s, 3.6 and 9.9 m/s, 5.2 and 6.0 m/s, 6.0 and 9.9 m/s, 7.5 and 9.9 m/s and 9.9 and 10.7 m/s. In conclusion, the degree of fiber orientation increases with increasing relative collector velocity for batch 1 as well as for batch 2 but the initial hypothesis of a linear relation was not supported by these results.

### 3.2. Mechanical Testing

**Figure 9:**
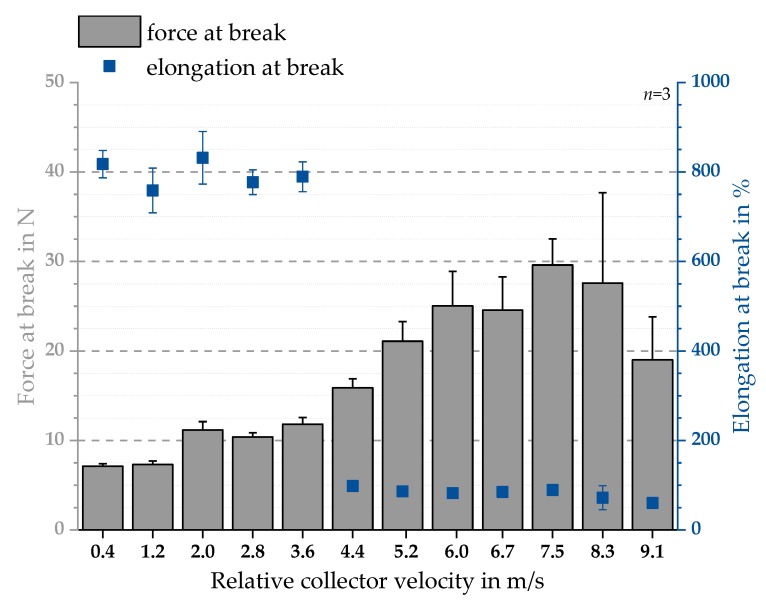
Uniaxial mechanical testing of samples of batch 1. Displayed are elongation at break in % (**blue squares**) as well as force at break in N (**grey bars**) (mean ± SD, see Equation (10)). The measured elongation at break displays a distinct decrease for all relative collector velocities ≥4.4 m/s.

We initially hypothesized a linear relationship between the relative collector velocity and the mechanical properties. To evaluate this hypothesis, uniaxial tensile tests were carried out for PCL fiber scaffolds. The elongation and force at break were measured for each relative collector velocity. As shown in Figure 9, elongations at break around 70% and 800% were found. Forces at break were between 9.87 N and 36.4 N. In addition, a distinct difference between the elongation at break for all relative collector velocities ≤3.6 m/s and ≥4.4 m/s was observed. Furthermore, forces at break increase up to 7.5 m/s. No linear relationship between relative collector velocity and force at break was found. Therefore, the initial hypothesis of a linear relation was not supported by the analysis results. At the same time, the assumed similar trend of the degree of fiber alignment and the mechanical properties was shown.

### 3.3. MM Measurements

**Figure 10:**
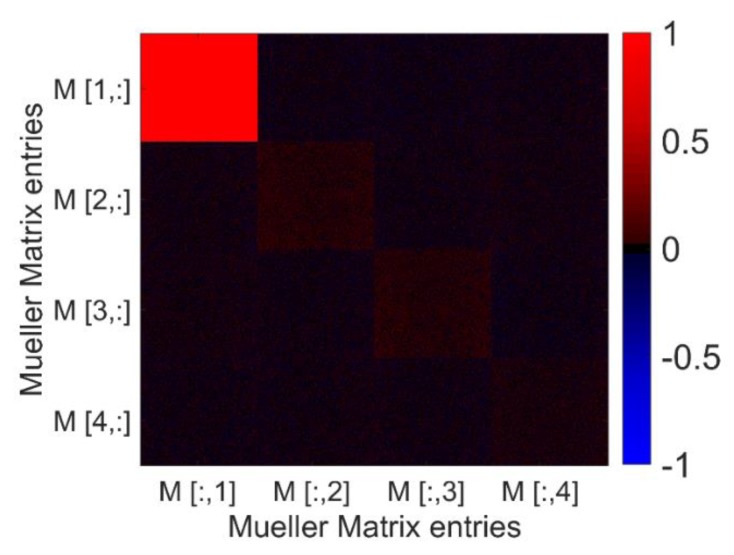
MM images of a fiber scaffold sample fabricated at a relative collector velocity of 0.4 m/s. The result is comparable to the MM of a diffuse scattering sample, indicated on the right side of Figure 5. The data shows that the sample is homogeneous.

A scheme of the result of an MM measurement is shown in the image matrix in Figure 4. If the sample is not homogeneous, different MM signals are found for the different locations (for an example see [40]). The fiber scaffolds in this work are homogeneous, appear white and exhibit diffuse scattering as can be seen in Figure 6a. The theoretical MM for a diffuse scattering sample is shown in the matrix on the right side in Figure 4. The measured MM for a fiber scaffold fabricated at a relative collector velocity of 0.4 m/s is displayed in Figure 10. The image matrix element *M*[1,1] has the value one for every pixel, as every image is normalized to it. As expected for a scattering sample, the other image matrix elements are small.

#### 3.3.1. MM for Different Spin Velocities in Transmission

**Figure 11:**
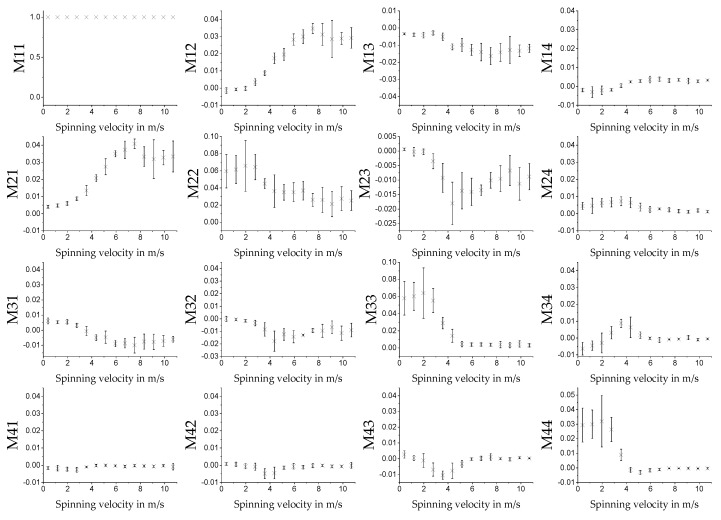
Average values of the MM images of the different MM entries for different spinning velocities. Measurement was performed in transmission with the 532 nm light source. The fibers of the sample were aligned parallel to the axis of vertical polarization of the MM measurement setup. The error bars show the standard deviations for the given sample size of *n* = 5.

**Figure 12:**
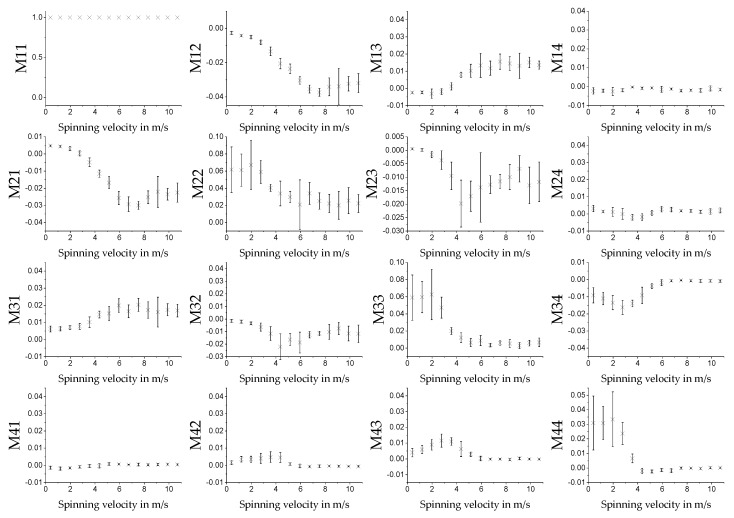
Average values of the MM images of the different matrix entries for different spinning velocities. Measurements were performed in transmission with the 532 nm light source. The fibers of the sample were orientated horizontally with respect to the orientation of the measurement setup. The error bars show the standard deviation for the given sample size of *n* = 5.

The measurement on the fiber scaffolds was performed for samples fabricated at 14 different spin velocities. Five different batches were produced on different days to verify the reproducibility of the measurement. The scaffolds were measured in transmission. Because homogeneity could be seen in the measurement results, see Figure 10, the MM was calculated over the average of all pixels in the images. The average MM values for different spinning velocities are displayed in Figure 11. The measurements were performed with the 542 nm light source. Other light sources were also used. The qualitative shape of the measurement results was the same, only the amplitude of the signal was different (see Supplementary Materials). For technical reasons, the green light source was selected for further measurements. The error bars display the standard deviation for the five different batches and are calculated using Equation (10). As known from Section 3.1, the fibers in the samples are aligned in one direction. The degree of alignment in this direction depends on the spinning velocity of the rotating collector. The direction of alignment is parallel to the rotation direction of the collector. For MM measurements, direction of alignment of the fiber scaffolds was aligned once parallel to the horizontal and in another run to the vertical (by rotating the sample by 90°) polarization state of the MM measurement system (see Section 2.2). Figure 11 shows the result for the vertical direction. The qualitative behavior for *M*12 and *M*21 is, for example, reversed when the samples are oriented horizontally in the spinning direction, see Figure 12. As can be seen for *M*12 and *M*21, the MM matrix element values increase for increasing spinning speed up to about 7 m/s. From there on, it stagnates, and the standard deviations increase for the different batches.

#### 3.3.2. Key Figures for Different Spin Velocities

**Figure 13:**
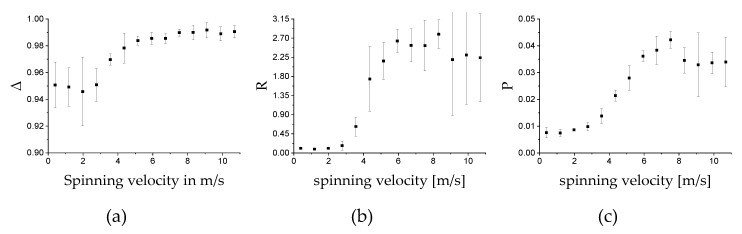
Key figures ∆ (**a**), *R* (**b**), and *P* (**c**) of the average values of the MM images of the different entries for various spinning velocities measured with 532 nm in transmission mode. The error bars show the standard derivation for the given sample size of *n* = 5.

The key figures ∆ (depolarization power), *R* (total retardance) and *P* (total polarizance) were calculated according to Equations (5)–(7) (see Figure 13). Calculation was performed for the average MM over all pixels imaging the sample and samples were manufactured with different spin velocities. Laser radiation at 532 nm was used for the measurement. The sample size is *n* = 5. From the calculation of the key figures, results show that there is no reversed trend observable, as is obvious for the M21 and *M*12 matrix element values in Figure 11 and Figure 12. Due to their calculation, the key figures are less susceptible to rotation of the sample.

#### 3.3.3. MM Information about Relative Orientation of the Fibers

**Figure 14:**
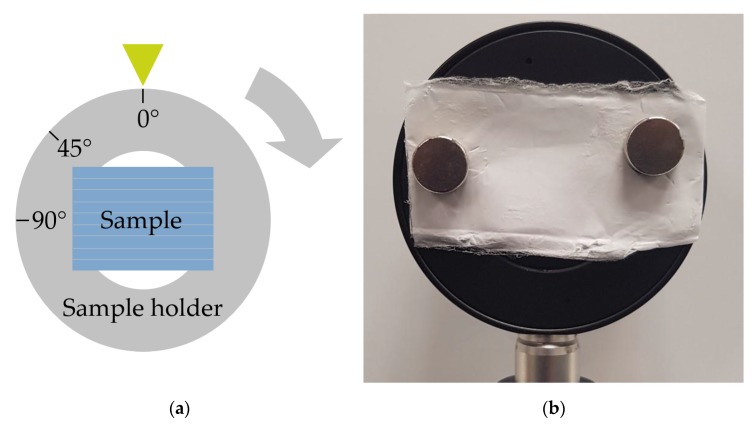
Front view of (**a**) a sketch of the sample in the sample holder and (**b**) a photo of the real sample in the holder. The structure in the sample displays the direction of orientation of the fibers. As known from SEM images, the fibers are orientated tangentially to the rotation direction of the cylinder, see Figure 1. The sample is then rotated by 360°.

**Figure 15:**
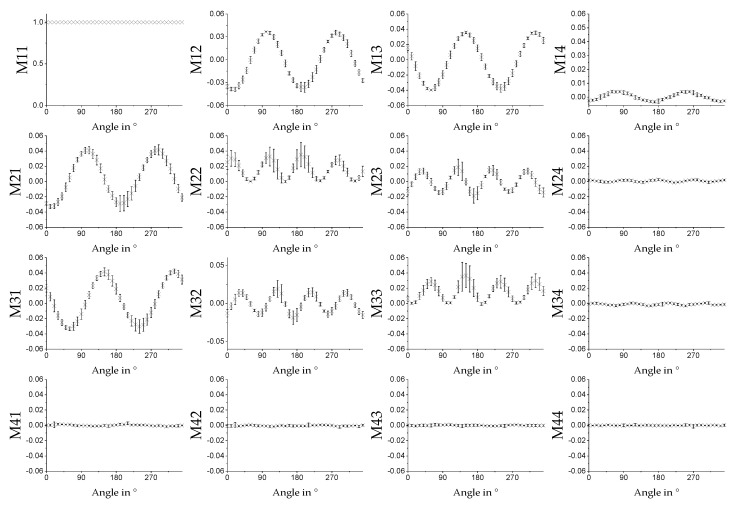
Average values of the MM images for spin velocity 7.5 m/s for different angles of the sample measured in transmission mode with the 532 nm laser. At 0°, the fibers of the sample are orientated horizontally. The error bars show the standard derivation for the given sample size of *n* = 5.

**Figure 16:**
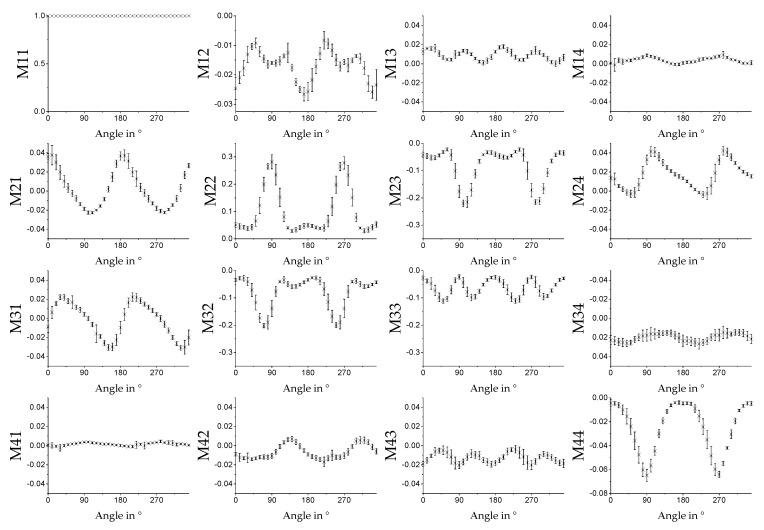
Average values of the MM images for spin velocity 7.5 m/s for different angles of the sample measured in reflection mode with the 532 nm laser. At 0°, the fibers of the sample are orientated horizontally. The error bars show the standard derivation for the given sample size of *n* = 5.

The MM also provides information on the orientation of the fibers relative to the experimental setup. As can be seen in Section 3.3.1 and Section 3.3.2, some MM entries and key figures vary as functions of the spinning velocity of the cylindric collector. The assumption is that this signal change correlates with the degree of alignment of the fibers. That is why we expect a periodic signal with period length of 180°, e.g., a sinusoidal dependence in the related MM entries for a measurement of a rotated sample, i.e., where the alignment changes direction. The concept of rotation and orientation of the sample in the measurement setup can be seen in Figure 14. In Figure 15, the average MM result for a measurement in transmission mode using fiber scaffolds (sample size of *n* = 5) spun with a spin velocity of 7.5 m/s and the 532 nm light source is shown. The sample was mounted on a rotation stage with magnets and then rotated in 10° steps. The same measurement was done in reflection mode. The result can be seen in Figure 16. In both Figure 15 and Figure 16, a strong angle dependence of some MM entries can be observed.

## 4. Discussion

The SEM image-based analysis of fiber alignment (described in Section 2.1.3) exhibits a strong dependence on the relative collector velocity. Similar to the measurements of fiber diameters (see Supplementary Material), high dispersion of values for fiber alignment was observed. For this reason, high susceptibility to error is indicated for the SEM-based analysis. Possible explanations for these results include the resolution of the SEM images and the manually measured orientation of the fibers. Nevertheless, with increasing relative velocity, the dispersion of the values decreases significantly until a relative collector velocity of 6.0 m/s to 7.5 m/s is reached. These results correlate with the data of the mechanical testing, which indicates a nonlinear dependence on the relative velocity.

**Figure 17:**
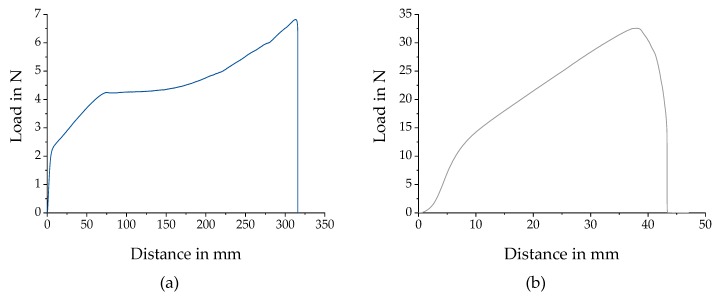
Exemplary curves of tensile tests. The load-distance curve for 0.4 m/s shows a section of constant load uptake (roughly 75 to 150 mm) following the initial load bearing (**a**). This indicates a macroscopic alignment of the fiber scaffold during external mechanical loads. The results for 7.5 m/s show an overall constant increase of load uptake for increasing distance until break (**b**).

In material sciences, a stress-strain curve is often used to compare the behavior of different materials to exterior mechanical influences. To generate comparable results, samples with defined geometric profiles, e.g., gauge lengths and cross-sections, are needed. In case of electrospun fiber scaffolds the reliable and nondestructive determination of the initial cross-section is extremely challenging, due to, for example, the highly porous structure of the scaffolds [11]. This hinders the calculation of stresses as characteristic values. Therefore, here, only force at break and elongation at break were plotted to visualize the mechanical properties and compare to the MM measurements. The generated results depend highly on the polymer, concentration, flow rate, process duration, dimensions of collector, relative velocity and environmental parameters used. Due to the influence of the relative collector velocity, the macroscopic behavior of the electrospun fiber scaffolds changes as well as the mechanical properties. For samples of batch 1, manufactured with relative velocities ≤6.0 m/s, an orientation of the fibers is assumed (see Figure 17a). After an initial load bearing a constant load, uptake during further stretching was observed (see Figure 17a). Subsequently, the recorded load increases until break. In contrast, the curves for relative velocities ≥ 6.9 show no plateau during tensile testing (see Figure 17b). This supports the hypothesis of an alignment of the macroscopic fiber structure as response to external mechanical loads within scaffolds with higher dispersion of fiber alignment (see Figure 17).

**Figure 18:**
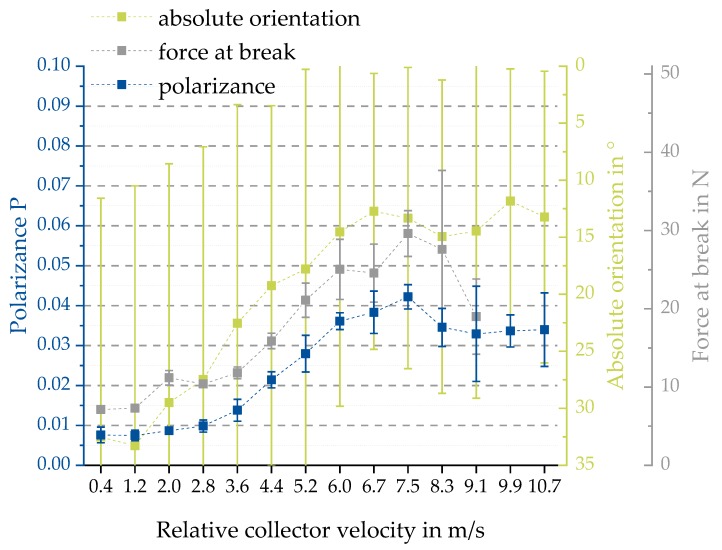
Correlation between absolute orientation measured with the image-based analysis, the polarizance P measured with the MM measurement system and the force at break. The blue squares show the results for the polarizance P (left Y-axis), the green squares represent the absolute degree of orientation of batch 2 and the grey squares show the results for the force at break of batch 1. Errors are the calculated standard deviations (see Equation (10)).

The MM data correlate with the manufacturing parameters of the fiber scaffolds. This is exemplified with the parameter of polarizance *P* or the MM entry *M*21. The characteristic shape of the experimental curves can be measured for a sample set, which was produced under fixed parameters such as concentration of the polymer solution, dimensions of the collector and for varying relative velocity of the drum collector. In Figure 18, the results for the polarizance, the absolute orientation measured with the image-based analysis and the mechanical data for the force at break from Section 3 are compared. Other than the boxplots in Figure 8, the average value of the angle divergence to the expected orientation is displayed in Figure 18. It can be seen that the qualitative trend for the measured polarizance, the orientation measured with the image analysis method and the force at break are similar. The error bars show that the precision of the MM measurement is higher than the precision of the image-based analysis method. However, the accuracy is difficult to evaluate due to the large error bars of the image-based analysis, which is the state-of-the-art measurement for fiber orientation so far. The values increase to a relative spin velocity of 6 m/s. Then the values increase to a velocity of approximately 7.5 m/s with a lower gradient. Subsequently, the values decrease to 9.1 m/s, where the dataset for the mechanics ends.

The results in Figure 11, Figure 12 and Figure 13 show that if the direction of the orientation of the fibers is known, one MM measurement is sufficient to determine the relative velocity at which a sample was produced. If both the orientation and the used relative velocity are unknown, three measurements are sufficient to determine the sinusoidal signal shape of, for example, the entry *M*21 of the MM image matrix, shown in Figure 15, and to read the relative velocity from its amplitude. In Figure 16, the entry *M*21 can also be used for this purpose. Here the signal does not exhibit a sinusoidal shape. 

**Figure 19:**
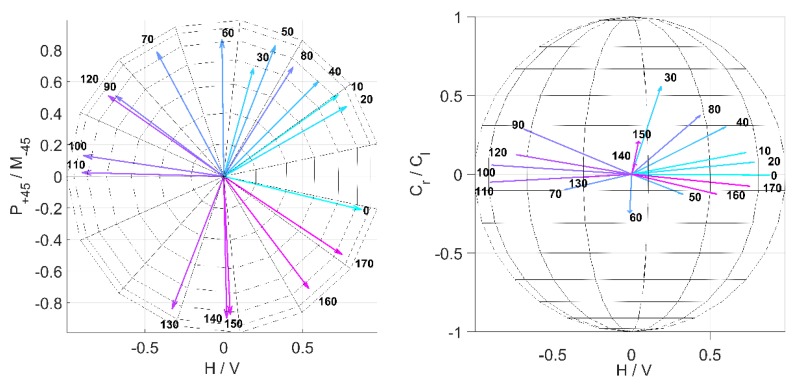
Normalized Stokes vectors for the fast axis of retardation, calculated from $M_{R}$, for different sample orientation (see Figure 14 and Figure 15) displayed in a Poincaré-sphere. The vectors point to the equatorial plane, which shows the orientation of the fast axis between 0° and 180°.

To obtain information about the orientation, further calculations can be performed, as shown in Figure 19. The normalized Stokes vector for the fast axis of retardation can be calculated from the retardance matrix $M_{R}$ (see Equations (8) and (10)), which is a result of the decomposition (Equation (3)) and displayed in a Poincaré sphere (see Figure 19). It can be seen that the vectors point to the equatorial plane on which the linear polarization states are represented with an orientation from 0° to 170°. The vectors are marked with the degree of orientation of the fibers in the sample as displayed in Figure 14, correlating with the results shown in Figure 15. A trend can be seen wherein the orientation of the sample is correlated with the calculated orientation of the fast axis, i.e., the axis of orientation of the light polarization with the largest propagation velocity in the material. The displayed data show the arithmetic mean for the 5 samples (each angle was measured twice, because the sample was rotated by 360°). The standard derivation of the angle between the ten vectors calculated and the average vector for the sample set is 48°. However, some vectors are not located on the equatorial plane. It seems that the measurement errors are correlated with the comparably higher standard deviation displayed in Figure 16 in the angle range from 100° to 140° and 170° and 220°. The comparably higher error could result from imprecise mounting of the sample to the rotating sample holder. However, the results show that it is possible to measure the direction of the fiber orientation in relation to the measurement coordinate system with a single measurement and without knowledge of the relative collector velocity.

The results of the MM measurements can also be interpreted in terms of the physical properties of the sample. The angle dependence of the signal shows that the amplitude of the signals in some of the MM entries depends on the amount of fibers which are aligned along that direction. That is why the signal is most likely to be correlated with the homogeneity of the fiber orientation within the fiber scaffold. The result of the decomposing suggests, that the fibers have diattenuation properties. Other studies additionally show the birefringence nature of polymer fibers under a polarizing-interference Pluta microscope [61].

Currently, the measurement time depends on the exposure time of the camera and takes about 15–20 s. The main restriction of the measurement time of the current system are determined by two points: (i) the exposure time of the camera and (ii) the switching speed of the LCR. There are cameras that have a more suitable frame rate. To take 36 images within one second, the illumination has to be bright enough to obtain the required information with such low exposure times. Also, speckle modulation in the time domain must be fast enough with respect to the exposure time. The drivers of the of LCR also limit the setup shown in Figure 4, probably due to limitations in the control speed, which is determined by the communication protocol. This could be improved by developing our own LCR controllers. The limitation of the LCR itself stems from its switching time of 15 ms (fall time) at room temperature. However, there are new LCRs that have faster fall and rise times. Fall times are typically much higher than rise times and show a temperature dependence, in addition, they are then in the range of 0.5 ms. The LCR technique used in this approach, in general, would allow fast switching times below 15 ms. In total, an MM measurement at below 1 sec appears to be feasible. In this case, a live examination of the surgical results of a transplantation of a fiber scaffold could be performed in future. If the intended application is defined and only a specific MM entry is of interest, it is possible to acquire only those images that are needed to calculate that specific MM entry. For example, if the relative velocity is the investigated parameter, the *M*21 entry could be measured and calculated. This would result in shorter measurement times without having to further reduce speckle formation.

## 5. Conclusions

In summary, we presented an MM measurement setup suitable for measuring the relative orientation of the fibers and the degree of orientation for electrospun fiber scaffolds. This is, in contrast to SEM imaging, a non-contact and non-destructive measurement technology which is demonstrated for the first time. Also, the measurement inaccuracy is relatively low compared to image analysis as presented in Section 2.1.3 and Section 3.1.

In the future, the MM measurement system could be used to determine the orientation of electrospun fiber scaffolds in real time. As an illustration, the described MM measurement system could be used during surgical procedures, in order to determine the orientation of graded implants on-site [24]. We showed that the approaches employed so far indicate a high susceptibility to error with regard to determining the fiber orientation through image analysis. The presented MM measurement shows promising results for a quick, spatially resolved, non-destructive and non-contact determination of fiber alignment with improved accuracy.

## 6. Patents

European patent “Method for the morphological characterization of fiber mats by polarimetry” pending (No. EP19197842.8).

## Figures and Tables

**Table 1:** Materials and supplies used during the manufacturing of the electrospun scaffolds.

Materials/Supplies	Specifications	Model	Source
Syringe	10 mL	Omnifix^®^ Luer Lock Solo	B. Braun Melsungen AG, Melsungen, Germany
Syringe pump		Fusion 200	Chemyx Inc., Stafford, TX, USA
Polyethylene tubing	1000 mm with 0.9 mL/m	Original Perfusor^®^ Line	B. Braun Melsungen AG, Melsungen, Germany
Blunt cannula	0.80 mm × 22 mm	Sterican	B. Braun Melsungen AG, Melsungen, Germany
High voltage supply			Matsusada Precision Inc., Shiga-ken, Japan
Electric motor		RE 16	IKA Werke GmbH Co. KG, Staufen im Breisgau, Germany
Polycaprolactone (PCL)	80 kDa		Sigma-Aldrich Chemistry Corporate, St. Louis, MO, USA
2,2,2-trifluoroethanol (TFE)	99.8%		abcr GmbH, Karlsruhe, Germany

**Table 2:** Summary of samples produced. Batch 1 was manufactured with a process time of 120 min, due to the required stability and fiber scaffold thickness for mechanical testing. Batch 2 was fabricated with a process time of 20 min, in order to increase sample size and enable transmission MM measurements. In each batch the sample size indicates the numbers of samples produced with the same production parameters on the same machine, but in different days, to verify the reproducibility. (MM = Mueller matrix measurements, IBA = SEM image-based analysis and MS = mechanical testing).

Spin Velocity in m/s	Batch	Sample Size	Measurements Performed		
			MM	IBA	MS
0.4	1	3		X	X
	2	5	X	X	
1.2	1	3		X	X
	2	5	X	X	
2.0	1	3		X	X
	2	5	X	X	
2.8	1	3		X	X
	2	5	X	X	
3.6	1	3		X	X
	2	5	X	X	
4.4	1	3		X	X
	2	5	X	X	
5.2	1	3		X	X
	2	5	X	X	
6.0	1	3		X	X
	2	5	X	X	
6.7	1	3		X	X
	2	5	X	X	
7.5	1	3		X	X
	2	5	X	X	
8.3	1	3		X	X
	2	5	X	X	
9.1	1	3		X	X
	2	5	X	X	
9.9	2	5	X	X	
10.7	2	5	X	X	

**Table 3:** The calculation of the specific MM entries out of image data. The different polarization states are explained by Equation (2). The first letter on the right side of the equation stands for the polarization state of the illumination and the second letter for that of the measured state.

Image Calculation for the MM Image Matrix			
M[1,1] = HH + HV + VH + VV	M[1,2] = HH + HV − VH − VV	M[1,3] = PH + PV − MH − MV	M[1,4] = RH + RV − LH − LV
M[2,1] = HH − HV + VH − VV	M[2,2] = HH − HV − VH + VV	M[2,3] = PH − PV − MH + MV	M[2,4] = RH − RV − LH + LV
M[3,1] = HP − HM + VP − VM	M[32] = HP − HM − VP + VM	M[3,3] = PP − PM − MP + MM	M[3,4] = RP − RM − LP + LM
M[4,1] = HR − HL + VR − VL	M[4,2] = HR − HL − VR + VL	M[4,3] = PR − PL − MR + ML	M[4,4] = RR − RL − LR + LL

## Supplementary Materials

The following are available online at https://www.mdpi.com/2073-4360/11/12/2062/s1: Supplementary Materials on crystallinity, orientation and diameter of batch 1, Fiber diameter for batch 2 and MM measurements for different wavelengths.
