# Supplementary material for: Mueller Matrix Measurement of Electrospun Fiber Scaffolds for Tissue Engineering
Source: Polymers (Basel). 2019 Dec 11;11(12):2062. doi: 10.3390/polym11122062 (PMC6960730; doi:10.3390/polym11122062)
Supplement: Supplementary file 1 [file polymers-11-02062-s001.pdf]

Supplementary Material

For the Article: Mueller matrix measurement of electrospun fiber scaffolds for tissue engineering

Content

This Supplementary Material contains additional measurements performed with the scaffolds presented in the main part. Crystallinity measurements were performed on the fiber scaffold samples. Also, diameters of the fibers for batch 1 and 2 were measured. MM measurements were also performed at different wavelengths of the light source.

1. Crystalinity

In order to create reproducible, tailor-made electrospun fiber scaffolds, many different properties have to be considered. So in addition to the morphological and mechanical properties, chemical, thermal and optical properties are of interest and under investigation as well. These properties are mainly determining the crystallinity of the processed polymer. The latter represents the relation between amorphous and crystalline regions within the fibers [1–7]. The crystal-forming is initiated, based on fundamental mechanical processes, i.e., polymer melt, evaporation of the solvent or mechanical stretching [2–6,8]. During the electrospinning process, evaporation of the solvent and mechanical stretching, due to the relative velocity of the collector, occur. Therefore, an increased crystallinity can be reasonably expected for an increased relative collector velocity. In addition, the increased crystallinity leads to increased force at break and a decreased elongation at break [9]. Differential scanning calorimetry (DSC) measurements were carried out by using an analysis device (DSC204 F1 Pheonix, Eric Netzsch GmbH & Co. Holding KG, Selb, Germany) connected to a cooling controller (CC 200, Eric Netzsch GmbH & Co. Holding KG, Selb, Germany). Five to twelve mg of sample material was placed in sealed alumina pans (Al 25µl, Eric Netzsch GmbH & Co. Holding KG, Selb, Germany) with pierced lids. These samples were exposed to a heat cycle. Each of these cycles started at room temperature and heated the sample up to 120 °C with a heat rate of 10°C/min. Thermal analysis software (Proteus Analysis, Eric Netzsch GmbH & Co. Holding KG, Selb, Germany) was employed to quantify the enthalpy of fusion [10]. The detected heat flow is then plotted against the temperature. Exemplary results are shown in Figure 1.

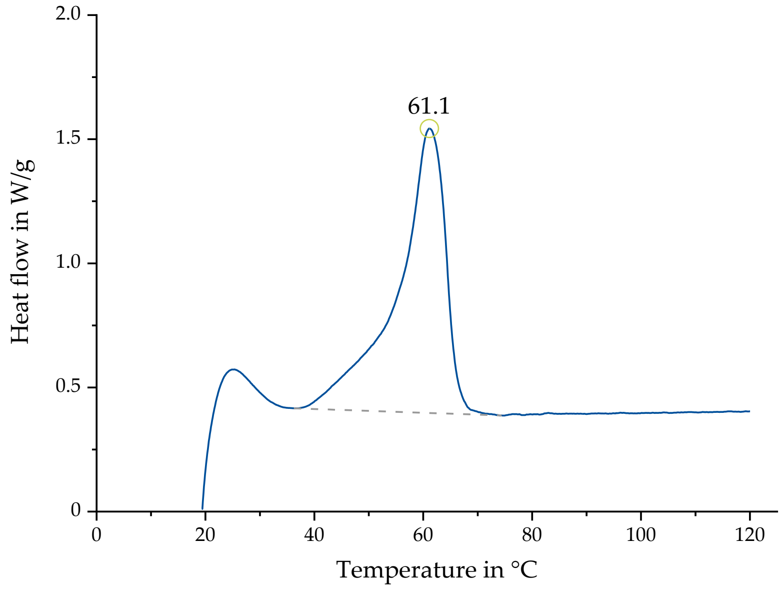

Figure 1. Exemplary data collected during DSC measurements. The area between the curve and the dashed base line represents the enthalpy of fusion and the peak of the curve (green cycle) the melting temperature (T_m). The depicted data is an example to further illustrate the used method

The degree of crystallinity ($\boldsymbol{X_c}$) was calculated using

$$X_c = \frac{\Delta H_F(T_m)}{\Delta H_F^0(T_m^0)} \quad (1)$$

with $\Delta H_F(T_m)$ being the detected enthalpy of fusion and $\Delta H_F^0(T_m^0)$ the enthalpy of fusion of completely crystalline PCL. In this case the used value for $\Delta H_F^0(T_m^0)$ was 139 J/g [11].

Figure 2 and Figure **3** show the different results for the crystallinity measurements for batch one and two. A possible explanation for the findings could be that the environmental parameters like humidity and temperature, which influence the evaporation rate, could influence the degree of crystallinity. The humidity ranged from 21% to 40% and the temperature from 22.4 °C to 26.3 °C. SEM images showed no "melted" or "fused", but clearly separated fibers. This indicates no critical influence of the detected humidity and temperature on the manufactured fibers. The DSC measurements do not support the initial hypothesis of a direct influence of the relative velocity on the crystallinity. Nevertheless, many different parameters have an impact on the crystallinity and this question needs further investigation.

The Differential scanning calorimetry (DSC) measurements for batch one show an increase of crystallinity with increasing relative collector velocity see Figure 2. Nevertheless no linear relationship between the relative collector velocity and the enthalpy of fusion could be established.

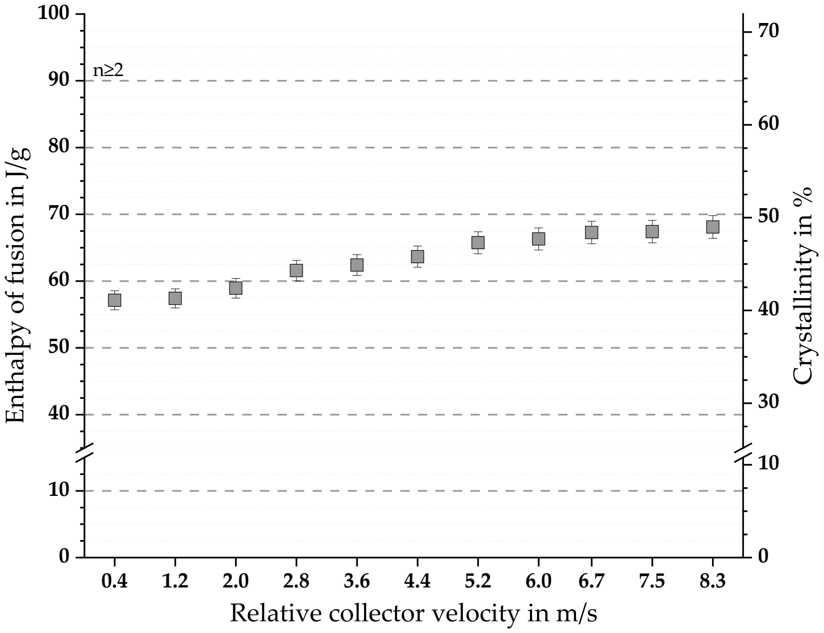

Figure 2. Enthalpy of fusion and crystallinity for batch one (mean ± measurement errors). The grey squares show the results for the enthalpy of fusion in J/g (left Y-axis) and the degree of crystallinity in % (right Y-axis) with error bars. With increased relative collector velocity an increased degree of crystallinity is observed.

In contrast to the DSC measurements for batch one (see Figure 2), the results for batch two display an increase of crystallinity for relative collector velocities ≤ 9.1 m/s and a decrease for relative velocities ≥ 9.9 m/s. Discrepancies between batch one and two can be explained by the influence of the electrospinning process. In addition, large standard deviations were calculated (see Figure 3). The unprocessed PCL was measured as a reference and the result is shown in Figure **3.** Enthalpy of fusion

and crystallinity for batch two (mean ±SD, see Eq. (10)). Blue squares show the results for the enthalpy of fusion in J/g (left Y-axis) and the degree of crystallinity in % (right Y-axis) with standard deviations. In this diagram, "0" represents the unprocessed PCL. With increased relative velocity an increased degree of crystallinity is observed, followed by a decrease. Therefore, a non-linear relationship between relative velocity and enthalpy of fusion was found. A Shapiro-Wilk test indicated normally distributed data sets. The following one-way ANOVA test resulted in no significant differences for the data sets.

labeled as "0". Despite the seemingly random results, statistical analysis was conducted. The Shapiro-Wilk test indicated normally distributed data sets. The following one-way ANOVA test showed no significant differences between the groups.

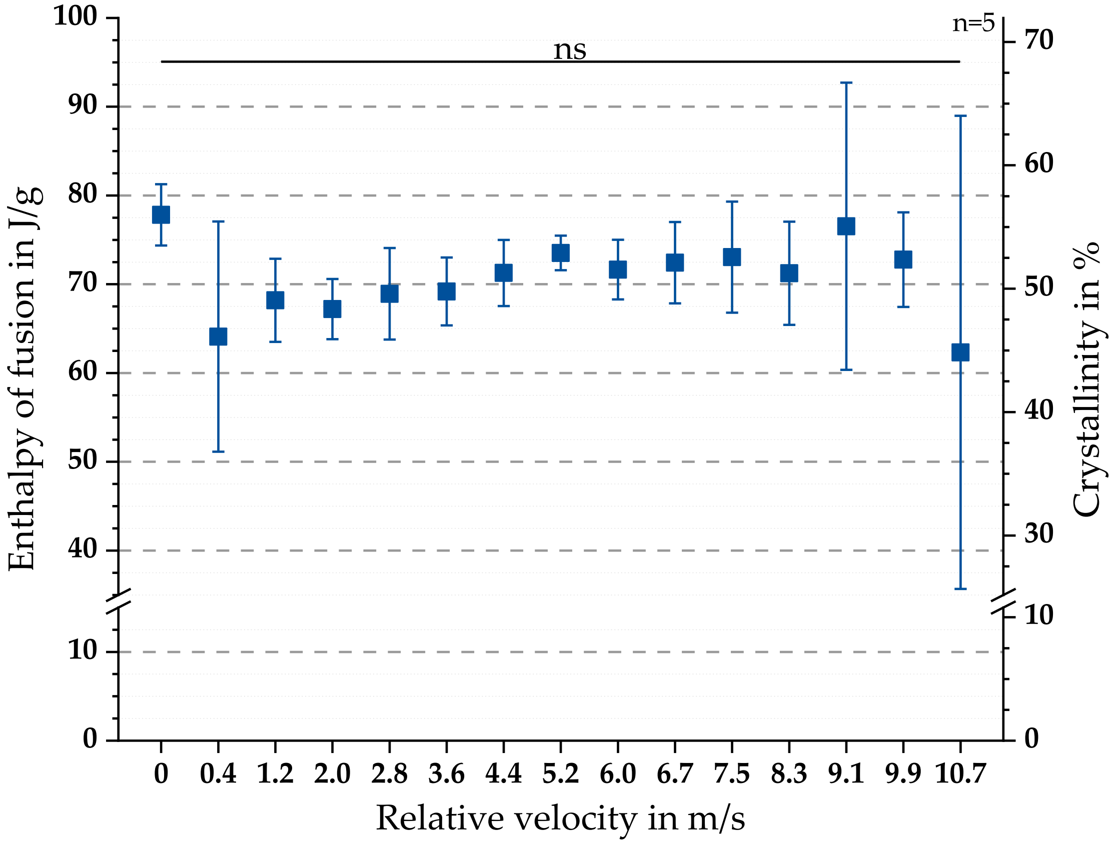

Figure 3. Enthalpy of fusion and crystallinity for batch two (mean ±SD, see Eq. (10)). Blue squares show the results for the enthalpy of fusion in J/g (left Y-axis) and the degree of crystallinity in % (right Y-axis) with standard deviations. In this diagram, "0" represents the unprocessed PCL. With increased relative velocity an increased degree of crystallinity is observed, followed by a decrease. Therefore, a non-linear relationship between relative velocity and enthalpy of fusion was found. A Shapiro-Wilk test indicated normally distributed data sets. The following one-way ANOVA test resulted in no significant differences for the data sets.

2. Orientation and diameter of Batch 1

To determine both, fiber diameters and fiber alignment, SEM (S-3400N, Hitachi High-Tech Analytical Science Ltd., Tubney Woods, Abington, UK) images of 3x15 s sputter coated (SC7620, Quorum Technologies Ltd., Laughton, East Sussex, UK) samples were taken. Five images for each of the 12/14 (batch 1/batch 2) samples manufactured at different relative collector velocities were recorded. Subsequently, the images were analyzed by using the image analysis software (AxioVision®, Carl Zeiss AG, Jena, Germany). For the determination of fiber diameters, a diagonal line was inserted splitting the

image in triangles. For every fiber crossing this line was measured three to five times on both sides of the line (see Figure 4 (a)).

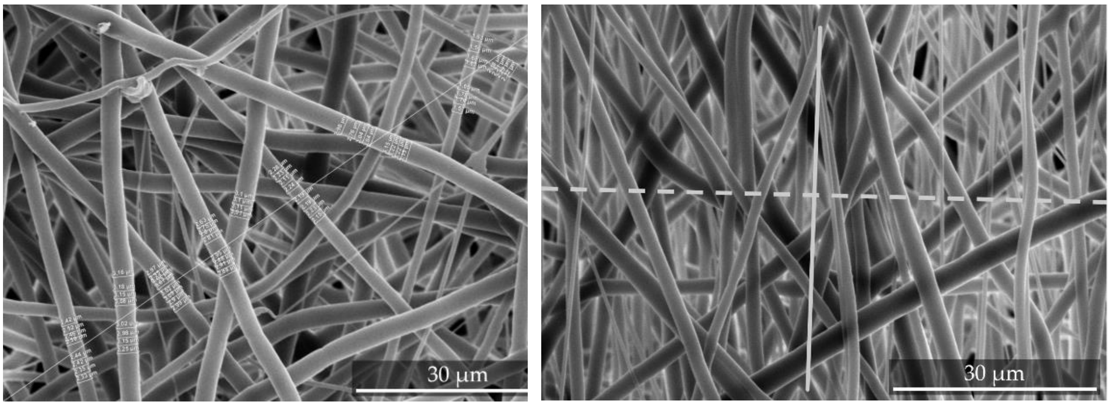

(a) (b)

Figure 4. Exemplary SEM images of electrospun fiber scaffolds. In order to determine fiber diameters each fiber crossing the diagonal line is measured three to five times on each side of the line (a). For the purpose of creating a reference for the analysis of fiber alignment, the solid line represents the moving direction of the collector (b). The second (dashed) line is perpendicular to the first line. To determine the orientation of each fiber, the angle between each fiber crossing the dashed line and this line is measured. The data shown is exemplary to further illustrate the used method.

As shown in Figure 5, fiber diameters from 0.3 μm up to 5.0 μm were measured for batch one. It was observed, that the dispersion of the values is varying with increasing relative collector velocity but generally the mean fiber diameters remain unchanged. To further address these observations, a Shapiro-Wilk [12] and a Kolmogorov-Smirnov [13,14] test were deployed. These tests showed normally distributed values throughout the groups. So in order to determine statistically significant differences, a one-way analysis of variance (ANOVA) was carried out. The results supported the initial observation of unchanged mean fiber diameters, because only for a velocity of 9.1 m/s significantly increased fiber diameters were discovered, in comparison to the case for 1.2 m/s (* p-value < 0.05).

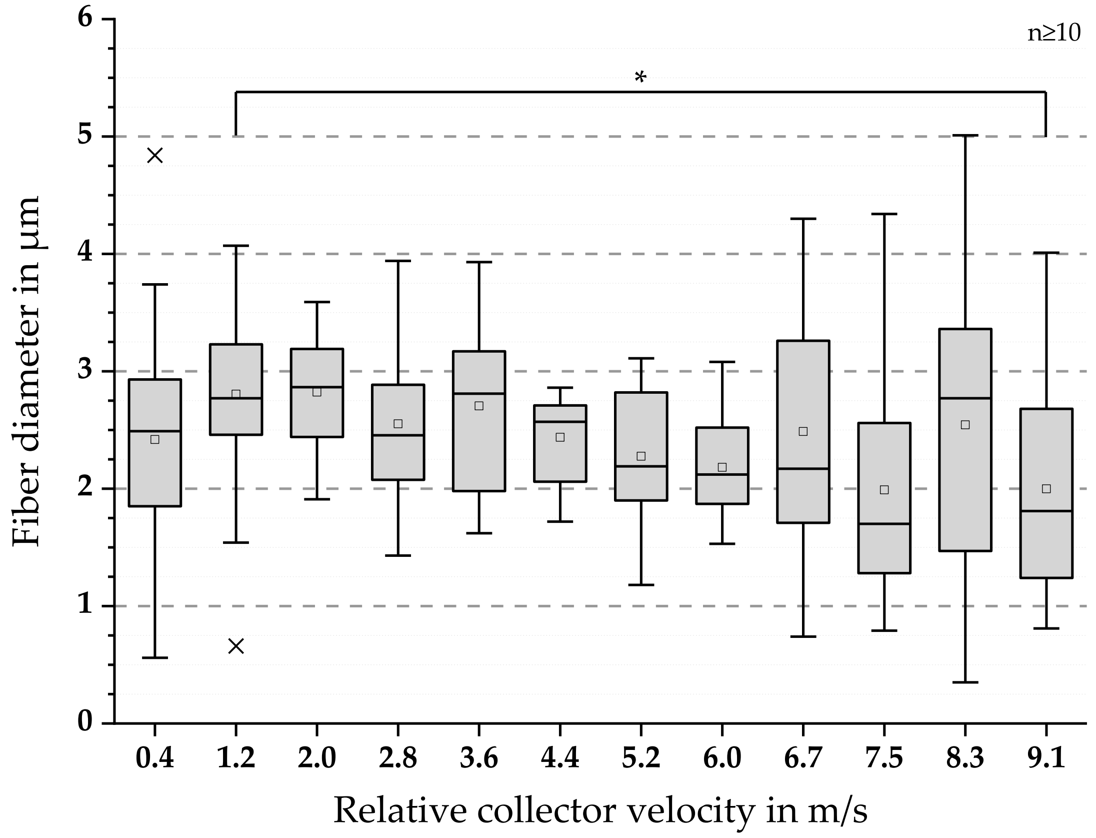

Figure 5. Boxplots of the fiber diameters in µm for batch one. The results show varying mean values without any noticeable trend. In order to check for a normal distribution, a Shapiro-Wilk and a Kolmogorov-Smirnov test were executed, with the result that the values are normally distributed. Therefore, an ANOVA test was performed. Significant differences were observed and labeled in the graph as following: * p-value < 0.05

3. Fiber diameter for Batch 2

The fiber diameters for batch two were investigated as well. The results are shown in Figure 6. The fiber diamters range from 0.33 µm to 5.49 µm and therefore show similar range of values as for batch one (see Figure 5). The results show also strong variations in dispersion of the values without observable trend for the mean values. In order to investigate statistical relations between the results for the different relative collector velocities, normal distribution was tested by Shapiro-Wilk and Kolmogorov-Smirnov tests. These tests showed not normally distributed values. Subsequently a Kruskal-Wallis ANOVA test followed by a Mann-Whitney test were conducted. These tests resulted in a vast amount of significantly different groups through out the relative collector velocities. Therefore only the non-significant groups were displayed and labeled "ns" (see Figure 6). The non-significant differences occurred for 1.2 m/s and 2.8 m/s, 4.4 m/s and 5.2 m/s, 6.0 m/s and 6.7 m/s, 6.0 m/s and 8.3/9.1/9.9/10.7 m/s, 6.7 m/s and 9.1/9.9/10.7 m/s, 7.5 m/s and 9.1 m/s, 8.3 m/s and 9.9 m/s, 9.1 m/s and 10.7 m/s and 9.9 m/s and 10.7 m/s.

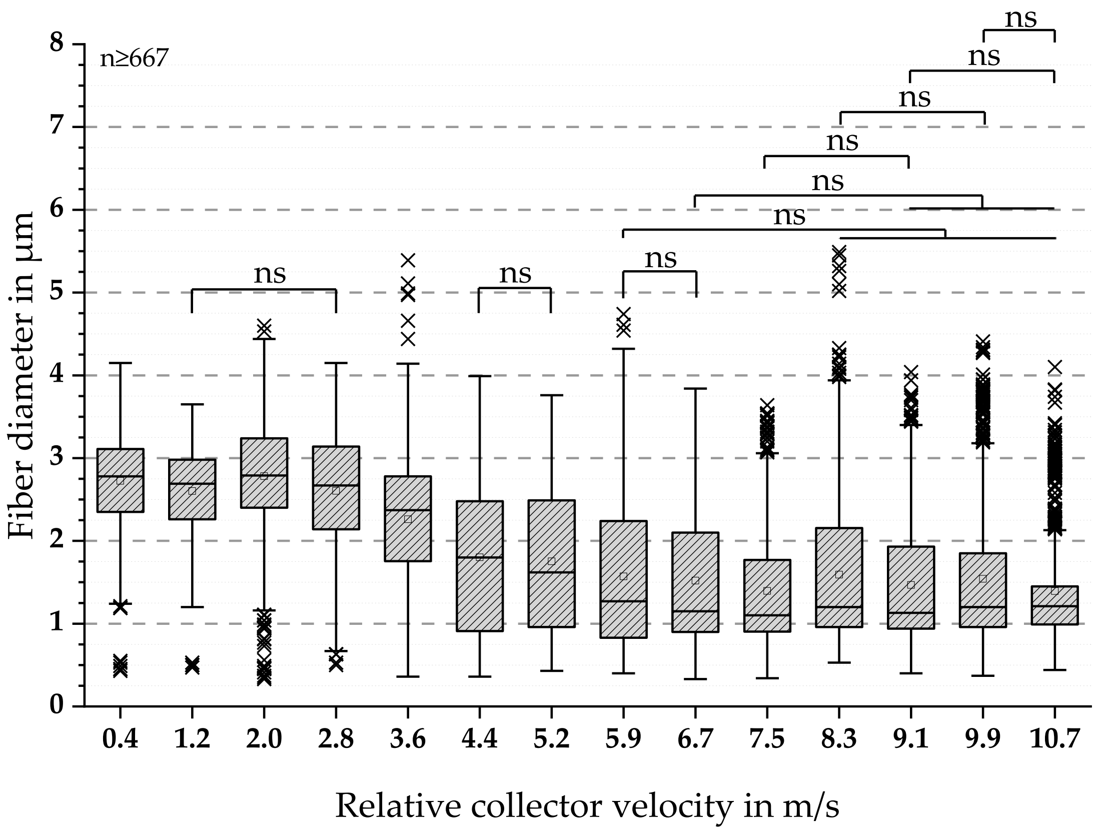

Figure 6. Boxplots of the fiber diameters in µm for batch two. The results for batch two show also varying mean values without any noticeable trend. In order to check for a normal distribution, a Shapiro-Wilk and a Kolmogorov-Smirnov test were executed, with the result that the values are not normally distributed. Therefore, a Kruskal-Wallis ANOVA test was performed, indicating significant differences in fiber diameters. In addition, non-parametric Mann-Whitnes tests were conducted. Due to the vast amount of significant differences between the groups, only the non-significant pairs are displayed and labeled with "ns".

The investigation of the morphology of the fiber scaffolds showed significant differences concerning fiber diameters for different relative collector velocities. The high dispersion of the values shown in Figure 5 and Figure 6 indicate a strong influence of the measurement method on the diameter. Despite the fact, that the protocol was developed to reduce errors based on measured fibers and image quality, it still needs further work. A possible solution would be the use of automated fiber diameter measurement [15].

4. MM-measurement for different wavelength

We also performed MM measurements with different wavelengths. The parameter of interest was the signal amplitude variation for different in spin velocities for the three wavelengths 445 nm, 532 nm, and 633 nm. The qualitative results of the measurements were the same for every wavelength. The result at a spin velocity of 7.5 m/s can be seen in Figure 7.

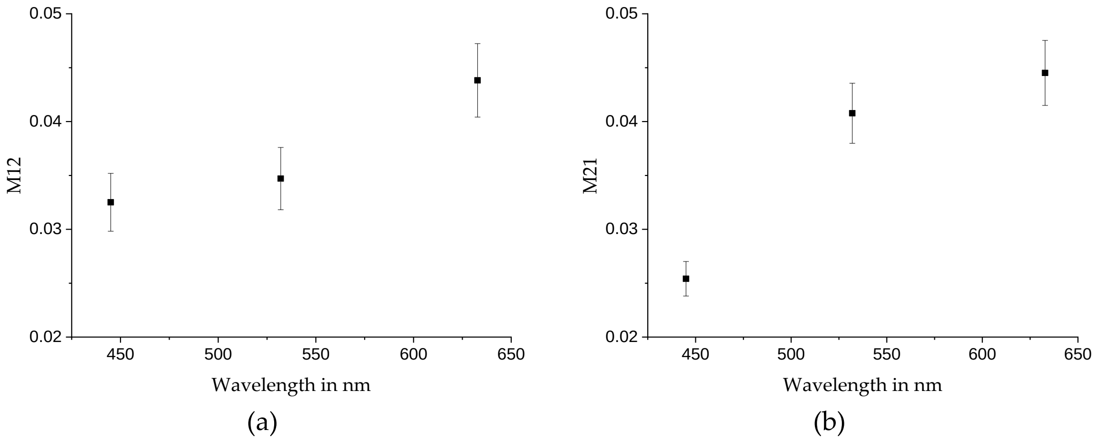

Figure 7. Average values of the MM elements M[1,2] (a) and M[2,1] (b) of fiber scaffolds fabricated with spin velocity of 7.5 m/s, measured with three different light sources at 445 nm, 532 nm, and 633 nm. The error bars represent the standard derivation for the given sample size of n=5.

References

1. C. B. Crawford and B. Quinn, "Physiochemical properties and degradation," in *Microplastic Pollutants* (Elsevier, 2017), pp. 57–100.
2. *Werkstoffkunde* (Vieweg, 2007).
3. C. E. Carraher and R. B. Seymour, *Polymer chemistry*, 6th edition revised and expanded. (M. Dekker, op. 2003).
4. J. Lehmann, "Die Beobachtung der Kristallisation hochpolymerer Substanzen aus der Lösung durch Kernspinresonanz," Kolloid-Z.u.Z.Polymere **212**, 167–168 (1966).
5. G. W. Ehrenstein, *Polymeric Materials. Structure, Properties, Applications (translated from German)* (2012).
6. L. C. Sawyer, D. T. Grubb, and G. F. Meyers, *Polymer Microscopy*, Third Edition (Springer New York, 2008).
7. D. Cassan, A. Becker, B. Glasmacher, Y. Roger, A. Hoffmann, T. R. Gengenbach, C. D. Easton, R. Hänsch, and H. Menzel, "Blending chitosan-g-poly(caprolactone) with poly(caprolactone) by electrospinning to produce functional fiber mats for tissue engineering applications," J Appl Polym Sci **94**, 48650 (2019).
8. N. Patil, L. Balzano, G. Portale, and S. Rastogi, "A Study on the Chain−Particle Interaction and Aspect Ratio of Nanoparticles on Structure Development of a Linear Polymer," Macromolecules **43**, 6749–6759 (2010).
9. A. Becker, D. de Cassan, H. Menzel, Y. Roger, A. Hoffmann, and B. Glasmacher, "Polycaprolactone:Chitosan-graft-Polycaprolactone blends enhance biocompatibility in electrospun scaffolds for tendon replacement. 46th ESAO Congress 3-7 September 2019 Hannover, Germany," Int J Artif Organs **42**, 460 (2019).
10. D. de Cassan, A. L. Hoheisel, B. Glasmacher, and H. Menzel, "Impact of sterilization by electron beam, gamma radiation and X-rays on electrospun poly-(ε-caprolactone) fiber mats," Journal of materials science. Materials in medicine **30**, 42 (2019).
11. A. Tiwari and B. Raj, eds., *Reactions and mechanisms in thermal analysis of advanced materials* (John Wiley & Sons; Scrivener Publishing, 2015).

12. S. S. SHAPIRO and M. B. WILK, “An analysis of variance test for normality (complete samples),” Biometrika **52**, 591–611 (1965).
13. G. Marsaglia, W. W. Tsang, and J. Wang, “Evaluating Kolmogorov's Distribution,” J. Stat. Soft. **8** (2003).
14. M. A. Stephens, “EDF Statistics for Goodness of Fit and Some Comparisons,” Journal of the American Statistical Association **69**, 730 (1974).
15. N. A. Hotaling, K. Bharti, H. Kriel, and C. G. Simon, “DiameterJ: A validated open source nanofiber diameter measurement tool,” Biomaterials **61**, 327–338 (2015).
